# Vertebral Fracture as a Predictor of Subsequent Extremity Fractures: A Systematic Review

**DOI:** 10.3390/jcm15072596

**Published:** 2026-03-28

**Authors:** Yousif Qais Al-Khafaji, Árpád Viola, Siran Aslan, Murtadha Qais Al-Khafaji, Mohamad Abdul Al, Mustafa Qais Al-Khafaji, Faris Ayasra, Shahad Qais Al-Khafaji, András Gati, Viktor Foglar, Mohammad Walid Al-Smadi

**Affiliations:** 1 Faculty of Medicine, University of Debrecen, 4032 Debrecen, Hungary; yousifqais7@hotmail.com (Y.Q.A.-K.); mohamad.abdulal.contact@gmail.com (M.A.A.); 2 Department of Neurosurgery, Dr. Manninger Jenő Traumatology Institute, 1081 Budapest, Hungary; fares.ayasra1@gmail.com (F.A.); shahadqais@gmail.com (S.Q.A.-K.); drgatiandras@gmail.com (A.G.); foglar.viktor@gmail.com (V.F.); smadi996@hotmail.co.uk (M.W.A.-S.); 3 Neurotraumatology Division, Semmelweis University, 1081 Budapest, Hungary; drsiran.5@gmail.com; 4 Doctoral School of Clinical Medicine, Semmelweis University, 1083 Budapest, Hungary; 5 Department of Trauma and Orthopaedics, Frimley Park Hospital, Frimley GU16 7UJ, UK; murtadha.al-khafaji1@nhs.net; 6 Department of Plastic Surgery, Norfolk and Norwich University Hospital, Norwich NR4 7UY, UK; mustafa.al-khafaji@nhs.net

**Keywords:** vertebral fractures, osteoporosis, fragility fractures, hip fractures, secondary prevention, fracture risk, bone density, aged, systematic review

## Abstract

**Background:** Vertebral fractures are the most common osteoporotic fractures and are frequently underdiagnosed. Although prior fragility fractures increase the risk of subsequent fractures, the magnitude and distribution of extremity fracture risk following vertebral fractures remain incompletely defined. **Objective:** The objective of this study is to evaluate the risk of subsequent extremity fractures following vertebral fractures in adults aged ≥50 years and to characterize fracture patterns and timing. **Methods:** A systematic review was conducted using three databases (PubMed, OVID, and Scopus) covering studies published between January 2005 and December 2025. Studies reporting subsequent extremity fractures after an index vertebral fracture in adults aged ≥50 years were included. Data extraction included patient demographics, fracture characteristics, treatment variables, and incidence of subsequent fractures. **Results:** Eight studies were included in the qualitative (narrative) synthesis, comprising a total of 488,770 patients with an index vertebral fracture. The reported incidence of subsequent extremity fractures ranged from 1.4% to 12.4%, with a crude aggregated incidence of 6.90% (33,605 patients). Hip fractures accounted for 73.3% of extremity fractures, followed by forearm/wrist (11.8%), humerus (10.3%), and ankle fractures (3.26%). Most subsequent extremity fractures occurred within 1–3 years after the index vertebral fracture. Additionally, 23,542 patients (4.82%) experienced subsequent vertebral fractures. Rates of dual-energy X-ray absorptiometry utilization and pharmacologic treatment ranged from 5% to 34.5%. **Conclusions:** Vertebral fractures in adults aged ≥50 years are strong predictors of subsequent extremity fractures, particularly hip fractures, with risk concentrated in the early post-fracture period. These findings support the concept of a systemic fracture cascade and emphasize the need for early detection and structured secondary prevention strategies.

## 1. Introduction

Fragility fractures are highly prevalent among adults aged ≥50 years and represent a major global health burden. Osteoporosis, a systemic skeletal disease characterized by reduced bone strength and impaired microarchitecture, is the principal underlying cause. Although most commonly associated with postmenopausal women, osteoporosis also affects a substantial proportion of older men [1,2]. Reduced bone mineral density (BMD) increases skeletal fragility and predisposes individuals to fractures under low-energy or physiological loading conditions [3,4].

Hip and vertebral fractures are traditionally regarded as the hallmark osteoporotic injuries [5], but extremity fractures occur more frequently and contribute significantly to disability, healthcare utilization, and mortality [6]. Globally, approximately 37 million fragility fractures occur annually among individuals older than 55 years [7], and osteoporosis affects nearly 500 million people worldwide according to World Health Organization criteria [8].

Vertebral fractures are the most common osteoporotic fractures across populations [9,10,11]. Despite their prevalence, many vertebral fractures are clinically silent and remain undiagnosed in routine practice [12,13]. This underrecognition leads to missed opportunities for secondary prevention, even though vertebral fractures reflect systemic skeletal fragility rather than isolated spinal injury.

A prior fragility fracture is a well-established and powerful predictor of future fracture risk [14,15]. Importantly, fracture risk is highest in the first one to two years after the initial event, a phenomenon described as “imminent fracture risk” [16]. This temporal clustering is particularly evident after vertebral and hip fractures and highlights the importance of early identification and intervention [17].

Emerging evidence indicates that vertebral fractures are associated with an increased risk of subsequent non-vertebral fractures, including extremity fractures [18,19]. Vertebral fractures predict future fractures independently of BMD, underscoring their role as robust clinical risk markers beyond densitometric measurements alone [20].

There is a rising trend in extremity fractures of osteoporotic origin, with a 30–46% increase in upper extremity fractures [21], and a projected increase in hip fracture incidence to nearly double from 2018 to 2050, owing to the ageing population [22]. Several studies report at least a 1.6-fold increase in extremity fracture risk among patients with prior vertebral fractures, and some suggest that the risk of subsequent fracture may be greater following an index vertebral fracture than after an index extremity fracture [23,24]. Furthermore, patients with osteoporosis and previous vertebral fractures demonstrate elevated short-term refracture risk within two years [25].

Despite these observations, the magnitude and distribution of extremity fracture risk following vertebral fractures remain incompletely defined. Reported estimates are heterogeneous, and the prognostic impact of clinically silent vertebral fractures is not fully clarified. A comprehensive synthesis of available evidence is therefore necessary to better characterize this association and inform secondary prevention strategies.

The aim of this systematic review is to evaluate the risk of subsequent extremity fractures following vertebral fractures in adults aged ≥50 years and to analyze the influence of relevant clinical variables on this relationship.

## 2. Materials and Methods

The Prospective Register of Systematic Reviews (PROSPERO) (ID: CRD420251275643) was used to register this systematic review, and we followed the PRISMA 2020 checklist to guide the methodology and reporting process (Table S1). A comprehensive electronic search of PubMed, OVID, and Scopus databases was conducted for studies published between the earliest available date and December 2025.

### 2.1. PICOs and Research Strategy

**Figure 1:**
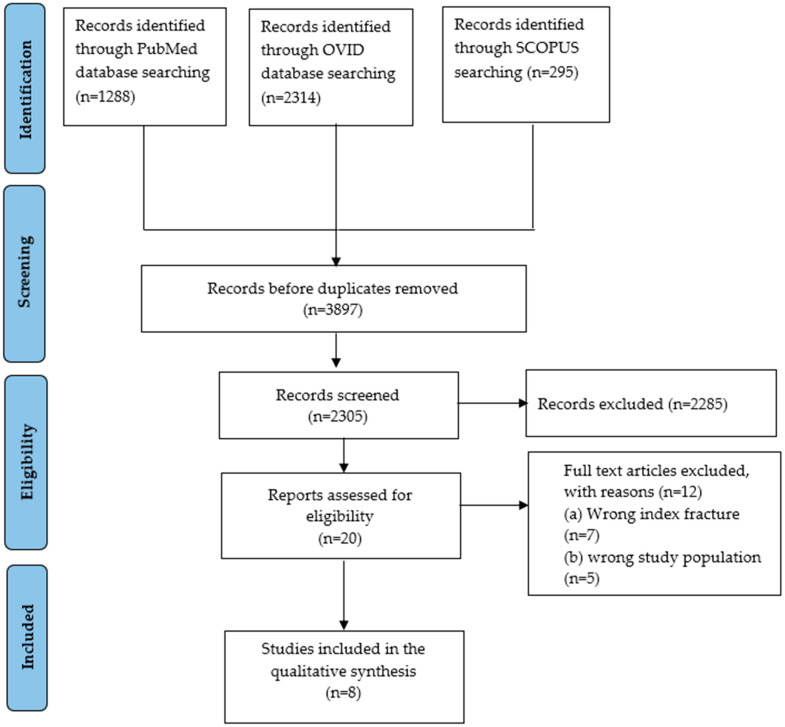
Schematic representation of study selection based on PRISMA.

The studies included in this systematic review were selected using an adapted Preferred Reporting Items for Systematic Reviews and Meta-Analyses (PRISMA) framework, as depicted in Figure 1. We utilized the PICOS framework (P: population; I: intervention; C: comparator; O: outcomes) to structure our analysis:

- P (Population): Adults aged 50 or older with a confirmed vertebral fracture (diagnosed radiographically or clinically).
- I (Intervention): Index vertebral fracture. This includes osteoporotic, traumatic, or pathological fractures serving as the exposure event for the “fracture cascade”, treated either pharmacologically or surgically.
- C (Comparison): No comparator group was included in the primary studies. However, the review focuses on incidence within the exposed population (patients with vertebral fractures). The absence of comparator groups in the included studies is acknowledged as a limitation.
- O (Outcomes): incidence of subsequent extremity fractures and quantify time intervals between index vertebral fracture and subsequent extremity fracture, to assess clinical outcomes (e.g., morbidity, mortality, functional status), to evaluate the influence of osteoporosis, pharmacotherapy, and demographic factors on risk.

### 2.2. Selection Criteria

The research team created inclusion and exclusion criteria collectively to ensure accurate and reliable study selection and data collection. Prospective and retrospective cohort studies, case–control studies, registry analyses, large administrative database studies, and randomized trials, if relevant fracture outcomes are reported, are included. Exclusion criteria: Editorials, comments, protocols, guidelines, conference abstracts, grey literature, anatomical, animal, cadaveric, or technical-only papers without clinical outcomes, non-English language scientific articles, and articles for which the full text was unavailable. Date restrictions were from January 2005. Furthermore, articles that did not address the research topic were removed.

### 2.3. Data Extraction and Management

Data extraction was performed independently by two reviewers using a standardized data collection sheet. Extracted variables included author name, database, journal, publication year, study design, DOI, population characteristics, fracture type, trauma mechanism, osteoporosis status (including dual-energy X-ray absorptiometry [DXA] T-score and diagnostic threshold), characteristics of the index vertebral fracture, treatment of the vertebral fracture, anatomical site of subsequent extremity fracture, time to extremity fracture, type of subsequent fracture, and occurrence of subsequent vertebral fracture.

Title and abstract screening were conducted independently by two reviewers. Full-text eligibility assessment was also performed independently by two reviewers. Discrepancies at any stage were resolved through discussion, and when necessary, adjudicated by a third reviewer.

### 2.4. Analysis and Synthesis of Data

A narrative summary was prepared for the studies included in the review based on the incidence and predictive factors of subsequent fracture after vertebral fracture, Complication rates, Patient survival, characteristics of index and subsequent fractures, and Recovery time. Any study that offered data outside of these categories was classified as miscellaneous and summarized in the text that followed. Due to heterogeneity in follow-up duration, fracture ascertainment methods, and outcome definitions across studies meta-analysis was not performed.

### 2.5. Assessing the Risk of Bias

Risk of bias (ROB) for all 8 studies was evaluated using the 2016 version of the ROBINS-I tool as described by Sterne et al. [26]. All the included studies were retrospective or prospective observational cohorts. Using the tool, the total risk of bias ranged from moderate to serious, due to the possibility of residual confounding in observational studies. Exposure and outcome classification raised concerns across most studies, due to reliance on administrative ICD classification, which may lead to misdiagnosis for vertebral fractures. However, most research used large samples amongst the population, which increased statistical power and generalizability of the results.

### 2.6. Evaluation of the Studies

Following abstract screening, a full-text screening was carried out using Microsoft Excel (v.16.73, Microsoft, Washington, DC, USA). This included the basic study information (title, author, publication year, link to full-text, and eligibility criteria). Each study was evaluated based on how well it met the inclusion criteria.

### 2.7. Structure Overview

In this systematic review, studies are categorized into two primary domains: (1) the incidence of subsequent extremity fractures following an index vertebral fracture in adults aged ≥50 years, and (2) predictors and risk factors influencing this progression within the fracture cascade framework. Incidence results are categorized by extremity fracture site (hip, humerus, forearm; upper vs. lower limb), sex-specific analyses, and time intervals between the index vertebral fracture and subsequent fracture. Predictor analyses are classified into risk factors, which include demographic factors, bone health measures (such as bone mineral density), past fracture history, comorbidities, pharmacotherapy, and other clinical characteristics. Studies are further classified by design and cohort characteristics to account for methodological variation and variability in risk estimates across populations.

## 3. Results

### 3.1. Study and Patient Demographics

**Figure 2:**
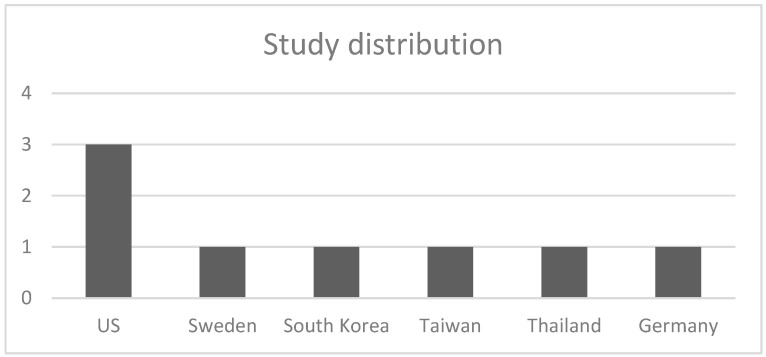
Studies based on the country of origin.

The database search identified 3897 records (PubMed: 1288; OVID: 2314; Scopus: 295). After duplicate removal and screening, 8 studies met the inclusion criteria (Figure 1). The included studies comprised retrospective database analyses, retrospective cohort studies, and prospective cohort studies conducted across multiple countries (Figure 2).

Publication years ranged from 2015 to 2024, with most studies published after 2018. A total of 488,770 index vertebral fractures were reported. Among them, 33,605 (6.90%) sustained a subsequent extremity fracture, and 23,542 (4.82%) sustained a subsequent vertebral fracture.

Only three studies reported detailed demographic data. In these cohorts, the mean age was approximately 72 years, and females accounted for 70.5% of patients. All included participants were aged ≥50 years [27,28,29]. Comorbidities were variably reported. Four studies used the Charlson Comorbidity Index, and one used the American Society of Anesthesiologists (ASA) score [27,28,29,30,31]. Reported conditions included cardiovascular disease, hypertension, diabetes, osteoarthritis, respiratory disease, liver and renal disease, dementia, depression, and malignancy. Two studies did not report comorbidity data [32,33].

### 3.2. Fracture Diagnostics and Characteristics

Most studies included vertebral fractures at all spinal levels, whereas two studies specifically restricted inclusion to thoracolumbar fractures [32,33]. Chen et al. (2022) included only single index vertebral fractures [29], while Sriruanthong et al. (2022) included both single and multiple vertebral fractures as index events [32]. The remaining studies did not specify fracture multiplicity.

Fracture etiology was predominantly osteoporotic. Six studies explicitly defined index fractures as fragility fractures or osteoporosis-related fractures [24,27,28,31,32,33]. Lorentzon et al. (2024) included all vertebral fractures except pathological fractures [30]. Chen et al. (2022) did not specify fracture etiology [29].

Fracture identification and osteoporosis classification were primarily based on International Classification of Diseases coding systems, including ICD-10 (International Classification of Diseases, 10th Revision) codes [24,27,30,32] and ICD-9 (International Classification of Diseases, 9th Revision) codes [27,29,31,33]. Ha et al. (2015) identified vertebral fractures using radiographic and clinical assessment without specifying ICD coding [28]. Adams et al. (2022) applied a structured identification algorithm combining ICD diagnosis codes, procedural codes, physician Healthcare Common Procedure Coding System (HCPCS) codes, and visit-type data [27]. Chen et al. (2022) identified fractures through outpatient medical records [29].

Dual-energy X-ray absorptiometry (DXA) was reported in only one study [27]. In that cohort, 20.8% of participants underwent DXA assessment, and osteoporosis was defined using a T-score threshold of ≤−2.5.

Low-energy trauma was the most frequently reported injury mechanism [24,28,29,32,33]. Adams et al. (2022) also included non-traumatic fractures identified through algorithm-based criteria [27]. Weaver et al. (2017) did not specify trauma energy [31], whereas Lorentzon et al. (2024) included fractures across all trauma levels [30].

Treatment reporting was inconsistent. When described, management was primarily conservative and pharmacologic, consisting of anti-resorptive therapy. No study reported vertebroplasty, kyphoplasty, or surgical fixation for the index vertebral fracture. The most reported medications were alendronate and denosumab. Treatment rates ranged from 5% to 34.5% (Table 1). Several studies did not explicitly report pharmacologic management [28,29,33].

### 3.3. Primary Outcomes

All included studies confirmed at least one type of extremity fracture, following an index vertebral fracture. Of 488,770 patients who had an index vertebral fracture, 33,605 (aggregated incidence 6.90%) experienced a subsequent extremity fracture. However, individual study estimates varied substantially, ranging from 1.40% to 12.4%, reflecting heterogeneity in study design, follow-up duration, and fracture ascertainment. Of the extremity fractures, 24,622 (73.3%) were specifically hip fractures, 3976 (11.8%) were forearm/wrist, 3463 (10.3%) were humerus, and 1097 (3.26%) were ankle. 451 (1.34%) were classified as non-hip-non-vertebral (NHNV) without specifying the fracture site.

Most patients re-fractured between 1 and 3 years. However, one study reported subsequent fractures occurring up to 11 years post-index fracture [32]. The proportion of patients sustaining an extremity fracture after a vertebral fracture varied widely, from as low as 1.40% to over 12.4% in large population-based cohorts (Table 2). Overall, hip fractures were the most frequently reported extremity fracture across nearly all studies, consistently representing the largest proportion of post-vertebral fractures.

### 3.4. Secondary Outcomes

All studies except Ha et al. (2015) [28] reported a new vertebral fracture following an index vertebral fracture. This is because the study specifically investigated hip fractures following an index vertebral fracture. Data regarding secondary vertebral fractures are found in Table 3.

Of the 488,524 patients with index vertebral fractures, 23,542 (4.82%) experienced a subsequent vertebral fracture. The proportion of patients sustaining a new vertebral fracture ranged from 1.85% to 13.5%. Large population-based studies generally reported lower cumulative incidences, with rates between 2.80% and 5.40% within follow-up periods of one to three years [29,33]. In contrast, smaller cohorts or studies with longer follow-up reported higher proportions of new vertebral fractures, including one study in which more than half of patients experienced a subsequent vertebral fracture within three years. The timing of new vertebral fractures was most frequently reported within the first one to three years after the index fracture. One study with extended follow-up reported new vertebral fractures up to nine years after the index event, indicating a prolonged period of elevated vertebral fracture risk. Overall, these findings demonstrate that patients with an initial vertebral fracture remain at considerable risk for additional vertebral fractures.

## 4. Discussion

This systematic review demonstrates that vertebral fractures in adults aged ≥50 years function as powerful entry points into a systemic fracture cascade. Among 488,770 patients with an index vertebral fracture, 6.90% sustained a subsequent extremity fracture, and nearly three-quarters of these were hip fractures. Additionally, 4.82% experienced a subsequent vertebral fracture. These findings confirm that vertebral fractures are not isolated spinal events but markers of generalized skeletal vulnerability with measurable downstream consequences.

Importantly, although 6.9% may appear modest in absolute terms, this incidence exceeds expected background extremity fracture rates in comparable age-matched populations. Population-based studies have demonstrated that prior fragility fractures are associated with increased risk of subsequent fractures compared to individuals without prior fractures [34].

The predominance of hip fractures and the concentration of events within 1–3 years support the concept of imminent fracture risk. Prior registry studies have demonstrated that recent vertebral fractures significantly elevate short-term hip fracture risk [24,30]. Our pooled data reinforce this temporal clustering and extend the argument by quantifying the magnitude of the risk of extremity fracture following vertebral injury.

Prior studies have suggested that the fracture sequence may be bidirectional [23]. While our study only looked at vertebral fractures as the index event, it does not directly establish bidirectionality but rather aligns with this concept. Population-based analyses have shown that hip fractures predict subsequent vertebral fractures, underscoring the reciprocal relationship between axial and appendicular skeletal fragility. While earlier literature emphasized hip fracture as the initiating event, the present synthesis demonstrates that vertebral fractures are equally potent predictors of subsequent hip and extremity fractures. Together, these data support a systemic cascade model in which fragility fractures represent manifestations of global skeletal failure rather than isolated anatomical incidents.

Vertebral fractures possess distinctive characteristics that amplify their prognostic significance. They frequently occur with minimal trauma, are often clinically silent, and remain underdiagnosed. Yet they independently predict future fractures beyond bone mineral density alone. Failure to detect vertebral fractures may therefore obscure true fracture probability and delay intervention.

Most studies included in this review relied on administrative coding systems for fracture identification. Although suitable for large-scale epidemiology, such methods may underestimate the incidence of asymptomatic fractures or misclassify timing. Structured radiographic evaluation and vertebral fracture assessment during DXA scanning improve detection. Moreover, imaging research has demonstrated that integration of STIR-sequence MRI following CT enhances identification of acute vertebral fractures and improves differentiation between active and chronic lesions. T-score–guided imaging approaches further refine risk stratification and influence clinical decision-making by identifying instability and active bone edema.

Detection, therefore, is not merely diagnostic; it is prognostic. Fracture acuity, multiplicity, and stability may influence both immediate management and long-term fracture trajectory. Observational analyses of thoracolumbar fractures indicate that treatment modality and fracture stability are associated with survival outcomes, reinforcing the broader clinical implications of accurate vertebral fracture assessment.

Despite the strong predictive value of vertebral fractures, formal osteoporosis evaluation was inconsistently reported. Only one included study documented DXA use, and fewer than one-quarter of patients underwent densitometric assessment. Pharmacologic treatment rates ranged between 5% and 34.5%, highlighting a substantial gap between fragility fracture occurrence and implementation of secondary prevention.

This discrepancy is particularly concerning given that vertebral fractures often represent the first clinically recognized sign of systemic skeletal fragility. The low rates of densitometric assessment suggest that many patients may not undergo structured evaluation of fracture probability following the index event. Furthermore, metabolic and endocrine contributors to bone loss, such as vitamin D deficiency, hyperparathyroidism, thyroid dysfunction, renal impairment, or hypogonadism, may remain undetected if not systematically assessed. Failure to address these factors may partially explain the observed clustering of subsequent fractures.

The concentration of extremity fractures within the first three years after vertebral fracture represents a narrow but critical window for intervention. Missed diagnostic opportunities during this period may allow progression along the fracture cascade.

The finding that 73.3% of subsequent extremity fractures were hip fractures has profound implications. Hip fractures are associated with high mortality, functional decline, and institutionalization. Ha et al. reported particularly adverse outcomes among patients sustaining hip fractures with prior vertebral fractures [28]. The vertebral-to-hip trajectory, therefore, represents a high-risk pathway within the fragility spectrum.

Interpretation of refracture incidence must also consider competing mortality risk in elderly populations. Patients with vertebral fractures often carry a significant comorbidity burden, and mortality following major fragility fractures may attenuate the observed cumulative incidence of subsequent events. Therefore, the true biological propensity for refracture may be even greater than reported rates suggest, as some patients may die before sustaining or being documented with a subsequent fracture.

Although comorbidity indices such as the Charlson Comorbidity Index and ASA score were reported in several studies, they were primarily used descriptively, and no consistent stratified analyses were available to assess their impact on subsequent fracture risk.

Pharmacologic treatment was inconsistently reported across studies, and most did not provide stratified analyses evaluating its effect on subsequent fracture risk. Consequently, the impact of anti-osteoporotic therapy on reducing subsequent fractures could not be systematically assessed, representing an important limitation of the available evidence.

Biomechanically, vertebral fractures contribute to sagittal imbalance, kyphosis, impaired balance, and reduced muscular strength. These changes increase fall susceptibility and may explain the predominance of hip fractures observed across cohorts. Thus, the fracture cascade is driven not only by bone fragility but also by altered biomechanics and functional decline.

Incidence of subsequent extremity fractures varied between 1.40% and 12.4% across studies, likely reflecting differences in follow-up duration, trauma classification, treatment exposure, and fracture ascertainment methods. Larger population-based registries [30,33] provided broader generalizability, whereas smaller cohorts demonstrated higher cumulative incidence. All included studies were observational and subject to potential residual confounding. Administrative coding may underestimate asymptomatic vertebral fractures, and inconsistent reporting of fracture severity and baseline bone health limited deeper stratified analyses.

## 5. Conclusions

Vertebral fractures in adults aged ≥50 years represent critical inflection points within a systemic fracture cascade. They are strong predictors of subsequent extremity fractures, particularly hip fractures, with risk concentrated in the first 1–3 years. Accurate detection, structured post-fracture evaluation, and timely initiation of secondary prevention strategies are essential to interrupt this cascade and reduce the substantial morbidity and mortality associated with progressive skeletal fragility.

## Figures and Tables

**Table 1:** Reported treatments used, including the type, percentage of patients on medication, and the duration of use.

Study	Under Treatment	Type of Medications	% of Patients on Medication	Duration of Use
Lorentzon, et al. (2024) [30]	Yes	-	10.3	1 year
Adams et al. (2022) [27]	Yes	Alendronate (85%)	23.1	2 years
Weaver et al. (2017) [31]	Yes	Bisphosphonates or nonbisphosphonates	M-group: 11.7 C-group: 8.50 *	-
Ha et al. (2015) [28]	No	-	-	-
Chen et al. (2022) [29]	No	-	-	-
Sriruanthong et al. (2022) [32]	Yes	Alendronate Denosumab	5.00	-
Hadji et al. (2021) [24]	Yes	-	34.5	-
Dang et al. (2018) [33]	No	-	-	-

* M-/C-group: Medicare group and Commercial group, respectively. -: not explicitly reported.

**Table 2:** Primary outcomes; Incidence and characteristics of subsequent extremity fractures in patients with vertebral fractures.

Study	Country	*n* (VF)	Time to Fracture	*n* (Extremity Fractures)	Incidence (%)	Anatomical Location
Lorentzon, et al. (2024) [30]	Sweden	12,283	≤2 years	1522	12.4	Hip
Adams et al. (2022) [27]	USA	6572	≤1 year	92	1.40	Hip
Weaver et al. (2017) [31]	USA	15,264	≤1 year	Hip: 130	Hip: 0.85	Hip, NHNV **
				NHNV **: 451	NHNV **: 2.95	
				Total: 581	Total: 3.81	
Ha et al. (2015) [28]	South Korea	246 *	≤1 year	150	100 *	Hip
Chen et al. (2022) [29]	Taiwan	179,691	≤2 years	Hip: 4822	Hip: 2.68	Hip Humerus Wrist
				Humerus: 1304	Humerus: 0.76	
				Wrist: 2467	Wrist: 1.37	
				Total: 8593	Total: 4.78	
Sriruanthong et al. (2022) [32]	Thailand	595	Median: 37 months (range 0–129, IQR 18–60).	Hip: 31	Hip: 5.21	Hip Humerus Wrist
				Wrist: 8	Wrist: 1.34	
				Humerus: 1	Humerus: 0.17	
				Total: 40	Total: 6.72	
Hadji et al. (2021) [24]	Germany	9133	1 year	Hip/femur: 249	Hip/femur: 2.72	Hip/Femur Forearm Wrist Hand
				Forearm/wrist/ hand: 221	Forearm/wrist/ hand: 2.42	
				Total: 466	Total: 5.14	
Dang et al. (2018) [33]	USA	264,986	≤3 years	Hip: 17,626	Hip: 6.65	Hip Humerus Distal Radius Ankle
				Humerus: 2158	Humerus: 0.81	
				Radius: 1280	Radius: 0.48	
				Ankle: 1097	Ankle: 0.41	
				Total: 22,161	Total: 8.36	

* This study recruited patients with hip fractures and investigated whether they had a vertebral fracture before. ** NHNV fractures were those of the ankle or foot; clavicle; femur; tibia or fibula; wrist, hand, or forearm (radius and ulna); humerus; patella; pelvis; scapula; or ribs. *n*: number of fractures; VF: vertebral fracture.

**Table 3:** Secondary outcomes; incidence and characteristics of subsequent vertebral fractures in patients with vertebral fractures.

Study	New Vertebral Fractures (Yes/No)	Number of Patients with Index Vertebral Fractures	Number of Patients with New Fractures	New Fractures %	Time to New Vertebral Fractures
Lorentzon, et al. (2024) [30]	Yes	12,283	574	4.67	≤2 years
Adams et al. (2022) [27]	Yes	6572	184	2.80	≤1 year
Weaver et al. (2017) [31]	Yes	15,264	1811	11.9	≤1 year
Ha et al. (2015) [28]	No	-	-	-	-
Chen et al. (2022) [29]	Yes	179,691	5326	2.96	≤2 years
Sriruanthong et al. (2022) [32]	Yes	595	11	1.85	≤11 years
Hadji et al. (2021) [24]	Yes	9133	1229	13.5	Mean 223 days
Dang et al. (2018) [33]	Yes	264,986	14,407	5.44	≤3 years

## Data Availability

All data of this meta-analysis are available from the authors upon reasonable request.

## Supplementary Materials

The following supporting information can be downloaded at: https://www.mdpi.com/article/10.3390/jcm15072596/s1, Table S1: PRISMA 2020 checklist [35].
